# Supplementary material for: Actinomycetes Enrich Soil Rhizosphere and Improve Seed Quality as well as Productivity of Legumes by Boosting Nitrogen Availability and Metabolism
Source: Biomolecules. 2020 Dec 15;10(12):1675. doi: 10.3390/biom10121675 (PMC7765327; doi:10.3390/biom10121675)
Supplement: Supplementary file 1 [file biomolecules-10-01675-s001.pdf]

microorganisms

MDPI

Article

Actinomycetes improve seed nutritional quality and productivity of leguminous crops by boosting nitrogen availability and metabolism

Hamada AbdElgawad[1,2§,*], Walid Abuelsoud[3§], Sulaiman A. Al Yousef[4], Dalal Hussien M. Alkhalifah[5], Samy Selim[6], Gaurav Zinta[1,7], Mahmoud M. Y. Madany[3,8], Wael N. Hozzein[9,2,*]

1. Laboratory Integrated Molecular Plant Physiology Research, Department of Biology, University of Antwerp, Groenenborgerlaan 171, B-2020 Antwerp, Belgium; hamada.abdelgawad @uantwerpen.be (H.A.)
2. Botany and Microbiology Department, Faculty of Science, Beni-Suef University, Beni-Suef 62511, Egypt.
3. Department of Botany and Microbiology, Faculty of Science, Cairo University, Giza, P.O. 12613, Egypt; walidabc@gmail.com (W.A.)
4. Clinical Laboratories, Sciences Department, College of Applied Medical Science, Hafr Al Batin University, Hafr Al-Batin, Saudi Arabia; drsulaiman@uhb.edu.sa (S.A.)
5. Biology Department, College of Science, Princess Nourah Bint Abdulrahman University, Riyadh, Saudi Arabia; dhalkalifah@pnu.edu.sa (D.A.)
6. Department of Clinical Laboratory Sciences, College of Applied Medical Sciences, Jouf University, Sakaka, P.O. 2014, Saudi Arabia; abdulsalam@ju.edu.sa (S.S.)
7. Shanghai Center for Plant Stress Biology, Center of Excellence in Molecular Plant Sciences, Chinese Academy of Sciences, Shanghai 200032, China; gzinta@gmail.com (G.Z.)
8. Biology Department, College of Science, Taibah University, Al-Madinah Al-Munawarah, 41411, Saudi Arabia; madany@cu.edu.eg (M.M.)
9. Bioproducts Research Chair, Zoology Department, College of Science, King Saud University, Riyadh 11451, Saudi Arabia; whozzein@ksu.edu.sa (W.H.)

* Correspondence: whozzein@ksu.edu.sa (W.H.) + hamada.abdelgawad @uantwerpen.be (H.A.)

§ equal contribution as first author

Received: date; Accepted: date; Published: 15 December 2020

Table S1. Morphological characterization of the actinomycete strains isolated from the local legume fields. The sign + and – indicate presence or absence, respectively.

	**Isolates**	**1**	**2**	**3**	**4**	**5**	**6**	**7**	**8**	**9**	**10**	**11**	**12**	**13**	**14**	**15**	**16**	**17**
	**Aerial mycelium**	+	+	+	+	+	+	+	+	+	+	+	+	+	+	+	+	+
	**Pigmentation**	+	-	-	-	-	-	-	+	-	-	+	-	-	-	-	-	+
**Spore chain**	**Spiral**	+	+	-	-	+	+	+	-	-	+	+	+	-	-	+	+	+
	**Rectiflexibiles**	-	-	+	+	-	-	-	+	+	-	-	-	+	+	-	-	-
	**Verticillate**	-	-	-	-	-	-	-	-	-	-	-	-	-	-	-	-	-
**Substrate Mycelia**	**White**	+	-	-	+	-	+	-	-	-	-	-	+	-	+	-	+	-
	**Brown**	-	+	-	-	+	-	-	-	-	-	-	-	+	-	-	-	-
	**Yellow**	-	-	-	-	-	-	-	+	+	-	-	-	-	-	-	-	-
	**Orange**	-	-	+	-	-	-	-	-	-	-	-	-	-	-	-	-	-
	**Grey**	-	-	-	-	-	-	-	-	-	+	-	-	-	-	+	-	+
	**Red**	-	-	-	-	-	-	+	-	-	-	-	-	-	-	-	-	-
	**Violet**	-	-	-	-	-	-	-	-	-	-	+	-	-	-	-	-	-

Table S2. Biochemical characterization of the actinomycete isolates. The sign + and – indicate presence or absence, respectively.

	Isolates	1	2	3	4	5	6	7	8	9	10	11	12	13	14	15	16	17
N source utilization	L-Cysteine	+	+	+	+	-	+	+	+	+	-	+	+	-	-	+	+	+
	L-Phenylalanine	+	+	+	+	-	+	+	+	+	+	+	-	+	+	+	-	-
	L-Histidine	-	-	+	-	+	+	-	-	+	-	+	+	+	-	+	+	+
	L-Lysine	+	+	+	+	+	+	+	+	-	-	+	+	+	+	+	+	-
	L-Asparagine	-	+	-	+	-	+	-	+	-	-	+	+	+	+	+	-	+
	L-Arginine	+	+	+	+	+	+	+	-	+	+	+	+	+	+	+	-	+
	L-proline	+	-	+	+	+	-	+	+	-	+	-	+	+	-	+	-	-
	L-Valine	-	+	+	+	+	-	+	+	+	+	-	-	+	-	-	+	-
	Tyrosine	+	+	+	-	+	-	+	-	+	-	+	+	+	-	+	+	+
C source utilization	D-fructose	+	+	+	-	-	+	+	+	+	+	+	+	-	-	+	+	+
	D-glucose	-	+	+	+	+	+	-	-	+	-	+	+	+	+	+	-	-
	Sucrose	+	-	+	+	+	+	+	+	-	+	+	+	+	+	+	+	+
	Maltose	+	+	-	+	-	-	-	+	-	+	-	-	-	-	-	+	+
	Raffinose	+	+	+	+	-	+	-	+	+	+	+	+	+	+	+	+	+
	Lactose	-	+	+	-	-	+	-	+	-	-	+	+	+	-	+	+	-
	Galactose	+	+	+	+	-	-	+	+	+	-	-	+	+	-	+	+	-
	Meso-Inositol	-	-	-	-	+	+	-	+	+	-	+	+	-	-	-	+	-
	Cellulose	+	+	+	+	-	+	+	-	-	-	+	-	-	+	+	-	+
	Xylose	-	+	+	+	-	-	-	+	+	+	+	+	-	-	-	+	+
	Dextran	+	+	-	-	-	+	+	+	-	-	-	+	+	-	-	-	+
Enzymes activity	Catalase	+	-	+	-	-	+	+	+	+	-	+	-	-	+	-	-	-
	Peroxidase	-	+	+	-	+	-	-	+	-	-	-	+	+	-	+	+	+
	Starch hydrolysis	-	+	-	+	-	+	-	-	+	+	-	+	+	+	+	-	-
	Gelatin liquefication	-	+	-	+	+	+	-	+	+	-	-	+	-	+	+	-	+
	Casein hydrolysis	+	+	+	+	+	+	+	+	-	-	+	-	-	+	-	-	+
	Lipolysis	+	+	+	+	-	+	+	+	+	-	+	+	+	+	+	-	+
	Citrate utilization	-	-	+	+	-	+	-	-	+	-	-	+	+	+	-	+	+
	H_2S Production	-	+	-	+	-	+	-	+	-	-	-	+	-	-	+	+	+
	DNase	+	+	-	+	+	+	+	+	-	+	-	+	-	-	+	-	-
	Nitrate reduction	-	+	-	+	+	+	-	+	+	+	+	+	+	-	+	+	-
	Urease	-	+	+	+	+	+	-	+	+	+	+	+	+	-	+	+	+
	L- asparaginase	-	+	+	+	-	+	-	+	+	-	-	+	+	+	+	+	+
	L-glutaminase	-	+	-	+	-	+	-	+	-	-	-	+	-	-	+	+	+

Table S3. Analysis of physical and chemical properties of soils of soybean, kidney bean, chickpea, lentil and pea under control (cont.) and after enrichment with biologically active actinomycete isolates. Values are the average of three individual replicates (means ± S.D). meq/L = milliequivalents of solute per liter of solution. Different letters represent significant differences between the treatments in each crop (Duncan test; P < 0.05; n=4).

	EC ds/ml	pH	HCO3 (meq/L)	Ca^{++} (meq/L)	P (ppm)	K^{+} (meq/L)	Mg^{++} (meq/L)	Zn (ppm)	Cu (ppm)	T. Phen	Organic matter
Soybean-Cont	3.4±0.5a	7.9±0.5a	10.1±1a	13.8±1.1a	33.6±2.3a	2.7±0.3a	7.3±0.7a	11.2±1.1a	9.7±0.9a	112±6.3a	2.6±0.4a
Kidney Bean-Cont	3.5±0.3a	7.2±0.8a	10.2±0.2a	14.1±0.9a	33.1±2.1a	2.8±0.3a	6.9±0.6a	11.7±1.0a	9.8±1.1a	112±6.3a	2.6±0.4a
chickpea-Cont	3.3±0.2a	7.5±0.4a	9.9±0.7a	14.1±1.5a	35.2±2.3a	2.8±0.4a	7.5±0.4a	12.1±0.9a	9.4±1.4a	112±6.3a	2.6±0.4a
Lentil-Cont	3.4±0.4a	7.7±0.7a	10.1±0.8a	14.0±1.2a	34.7±1.9a	2.7±0.2a	7.1±0.4a	12.1±0.9a	9.9±1.7a	112±6.3a	2.6±0.4a
Pea-Cont	3.6±0.5a	7.8±0.8a	9.9±0.9a	13.2±1.2a	35.6±2.1a	2.6±0.4a	7.2±0.8a	11.8±1.0a	9.6±0.4a	112±6.3a	2.6±0.4a
Soybean-I2	3.3±0.4a	7.4±0.3a	10.5±1.1a	25.5±0.5b	46.2±3.2b	3.4±0.1b	11.2±0.9b	14.6±1.1b	10.6±0.8a	132±6.8b	6.6±0.4b
Kidney Bean-I2	4.2±0.3a	8.1±0.3a	9.3±0.9a	19.8±0.5a	31.2±0.5a	2.9±0.5a	7.6±0.8a	13.2±1.1b	9.9±1.2a	110±4.1b	7.1±1.5b
chickpea-I2	3.9±0.4a	7.7±0.7a	9.9±0.3a	13.5±0.8a	31.2±3.8a	2.7±1.4a	8.1±1.1a	14.2±1.1b	11.6±1.8a	141±5.3b	6.6±1.8b
Lentil-I2	3.7±0.3a	7.5±09a	13.2±1b	16.1±3.2a	39.2±2.2a	5.7±0.7b	9.6±2.5a	21.8±0.8b	16.9±1.1b	151±5.1b	16.9±1b
Pea-I2	4.1±0.4a	7.2±0.2a	15.5±2b	21.5±1.2b	42.6±7.1b	3.9±1.4a	12.2±0.7b	14.2±1.1b	17.4±1.4b	161±2.5b	5.4±1.9b
Soybean-I8	3.5±0.6b	7.2±0.5a	9.6±1.1b	18.7±1.1b	49.5±2.8b	4.1±0.8b	12.5±0.7b	26.5±1.2b	11.3±1a	133±4.4b	7.3±1.4b
Kidney Bean-I8	4.3±0.2b	8.1±0.9a	11.2±0.7a	15.6±2.1a	31.3±1.1a	2.6±0.7a	7.6±0.9b	17.3±1.1b	13±2.1a	117±3.1b	8±4.1b
chickpea-I8	3.4±0.4a	7.1±0.5a	8.9±0.7a	13.6±3.1a	39.3±1.5b	2.8±0.4a	7.6±1.1b	15.3±0.9b	12.1±1.9a	111±8.4a	12.129b
Lentil-I8	3.5±0.6a	8.1±0.3a	11.7±1.4b	15.3±3.1a	45.3±3.1b	7.4±1.1b	15.2±1.5b	27±1.15b	9.2±0.6a	178±3.1b	3.2±0.6b
Pea-I8	4.2±0.1b	8.1±0.7a	12.5±0.8b	17.7±3.7b	42.3±1.7b	6.2±1.7b	17.1±1.1b	21.9±2.5b	9.8±0.7a	176±5b	5.8±0.7b
Soybean-I12	3.7±0.8a	7.6±0.4a	14.2±1.9b	19.6±2.9b	49.5±1.3b	2.6±2.4a	14.6±0.9b	15.3±0.9b	13±1.9b	113±7b	4±0.1b
Kidney Bean-I12	2.5±0.3a	8.1±0.7a	10.9±1.3a	12.7±4.5a	51.3±1.9a	3.1±1.2a	11.5±0.8b	10.6±1.1a	12.1±1a	100±11b	6.1±1b
chickpea-I12	3.1±0.5a	7.2±0.8a	11.3±2.1a	21.6±0.2b	49.3±1.5b	3.4±1.1a	10.2±0.2a	12.8±1.2a	9.6±1.2a	164±4.1a	9.6±1.2b
Lentil-I12	3.2±0.5a	8.2±0.8a	15.8±0.8b	18.6±5.6a	65.3±3.5b	6.7±0.8b	10.2±1.6a	14.9±2.1a	11.1±0.8a	139±9.4a	4.1±0.8b
Pea-I12	3.1±0.4a	7.6±0.5a	14.5±1.1a	23.5±0.8b	62.3±1.7b	7.4±0.7b	15.2±2.9b	18.2±1.7b	13.6±1.1b	100±7.5b	9.6±1.2b
Soybean-I15	3.3±0.2a	8.1±0.9a	14.5±0.7b	23.6±2.9b	46.6±0.2b	2.4±0.2a	8.1±0.2a	10.6±2.2a	10±2.4a	83±5.9a	10±2.4b
Kidney Bean-I15	2.3±0.5a	7.2±0.3a	11.9±0.7a	14.1±1.7a	35.4±2.7a	3.0±1.7a	12±1.9b	11.1±1.1b	11±2.6a	96±8b	6±0.6b
chickpea-I15	3.2±0.8a	7.6±0.1a	12.5±0.6a	15.6±1.7a	35.6±2.6a	2.7±0.6a	9.4±0.3a	23.5±1.1b	12.3±1a	172±5.4b	2.3±0.3a
Lentil-I15	4.4±0.5b	8.1±0.7a	17.2±1.1b	26.1±4.1b	51.5±6.4b	5.3±0.5b	11.5±1.7b	17.5±1.8b	19.3±1.2b	161±5b	9.3±.3b
Pea-I15	4.7±0.3b	7.4±0.5a	20.7±1.8b	23.9±2.1b	47.6±0.2b	6.4±0.9b	11.1±0.2a	23.6±1.4b	10.3±0.4a	123±3.1a	4.3±0.2b

Publisher's Note: MDPI stays neutral with regard to jurisdictional claims in published maps and institutional affiliations.

© 2020 by the authors. Licensee MDPI, Basel, Switzerland. This article is an open access article distributed under the terms and conditions of the Creative Commons Attribution (CC BY) license (http://creativecommons.org/licenses/by/4.0/).
